# Poly[bis­[μ_2_-2-(2-pyridylmethyl­amino)ethanesulfonato]cadmium(II)]

**DOI:** 10.1107/S1600536809049162

**Published:** 2009-11-21

**Authors:** Zhong-Xiang Du, Xin-Hong Chang

**Affiliations:** a Department of Chemistry, Luoyang Normal University, Luoyang, Henan 471022, People’s Republic of China

## Abstract

The title compound [Cd(C_8_H_11_N_2_O_3_S)_2_]_*n*_, is a two-dimensional coordination polymer based on a Cd^2+^ atom and deprotonated 2-(2-pyridylmethyl­amino)ethanesulfonic acid (Hpmt). The complex has mol­ecular symmetry *C*_i_ as a consequence of the Cd^II^ being located on an inversion centre. Two N atoms of each pmt^−^ ligand coordinate to the Cd^2+^ ion and its sulfonate O atom bonds to an adjacent Cd^2+^ ion. 24-membered (–Cd—N—C—C—S—O–)_4_ rings are formed between neighbouring Cd^2+^ ions; these are inter­connected, forming a two-dimensional layer structure. In respect to stereogenic amino N atom and the inversion symmetry of the complex, the compound is a 1:1 racemate. The crystal packing is stabilized by inter­molecular N—H⋯O hydrogen bonds and further connected by π–π stacking inter­actions between the pyridyl rings [average inter­planar distance and centroid–centroid separation = 3.582 (1) and 3.634 (1)Å, respectively], forming a three-dimensional supra­molecular architecture.

## Related literature

For different coordination modes of the pmt^−^ ligand in complexes derived from Hpmt, see: Du & Zhang (2009 ▶); Li *et al.* (2006 ▶, 2007*a* ▶,*b* ▶, 2008*a* ▶,*b* ▶); Liao *et al.* (2007 ▶).
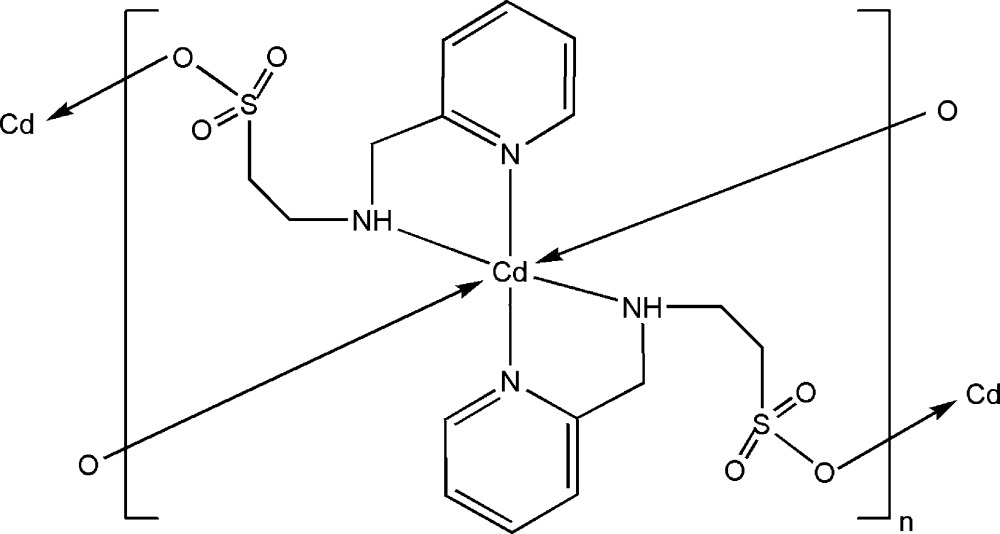


## Experimental

### 

#### Crystal data

[Cd(C_8_H_11_N_2_O_3_S)_2_]
*M* *_r_* = 542.90
Monoclinic, 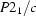

*a* = 8.8982 (8) Å
*b* = 14.0206 (12) Å
*c* = 7.9040 (7) Å
β = 103.579 (1)°
*V* = 958.52 (15) Å^3^
*Z* = 2
Mo *K*α radiation
μ = 1.40 mm^−1^
*T* = 291 K
0.22 × 0.16 × 0.12 mm

#### Data collection

Bruker APEXII CCD area-detector diffractometer
Absorption correction: multi-scan (*SADABS*; Sheldrick, 1996 ▶) *T*_min_ = 0.749, *T*_max_ = 0.849
5691 measured reflections
2178 independent reflections
2008 reflections with *I* > 2σ(*I*)
*R*_int_ = 0.011

#### Refinement

*R*[*F* ^2^ > 2σ(*F* ^2^)] = 0.019
*wR*(*F* ^2^) = 0.049
*S* = 1.03
2178 reflections
133 parameters
H-atom parameters constrained
Δρ_max_ = 0.48 e Å^−3^
Δρ_min_ = −0.38 e Å^−3^

### 

Data collection: *APEX2* (Bruker, 2004 ▶); cell refinement: *SAINT* (Bruker, 2004 ▶); data reduction: *SAINT*; program(s) used to solve structure: *SHELXS97* (Sheldrick, 2008 ▶); program(s) used to refine structure: *SHELXL97* (Sheldrick, 2008 ▶); molecular graphics: *SHELXTL* (Sheldrick, 2008 ▶); software used to prepare material for publication: *SHELXTL*.

## Supplementary Material

Crystal structure: contains datablocks global, I. DOI: 10.1107/S1600536809049162/kp2239sup1.cif

Structure factors: contains datablocks I. DOI: 10.1107/S1600536809049162/kp2239Isup2.hkl

Additional supplementary materials:  crystallographic information; 3D view; checkCIF report

## Figures and Tables

**Table 1:** Selected bond lengths (Å)

Cd1—N1	2.2853 (14)
Cd1—O2^i^	2.3496 (14)
Cd1—N2	2.3979 (15)

Symmetry code: (i) 
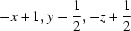
.

**Table 2:** Hydrogen-bond geometry (Å, °)

*D*—H⋯*A*	*D*—H	H⋯*A*	*D*⋯*A*	*D*—H⋯*A*
N2—H2⋯O3^ii^	0.91	2.26	3.089 (2)	152

Symmetry code: (ii) 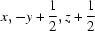.

**Fig. 1.**
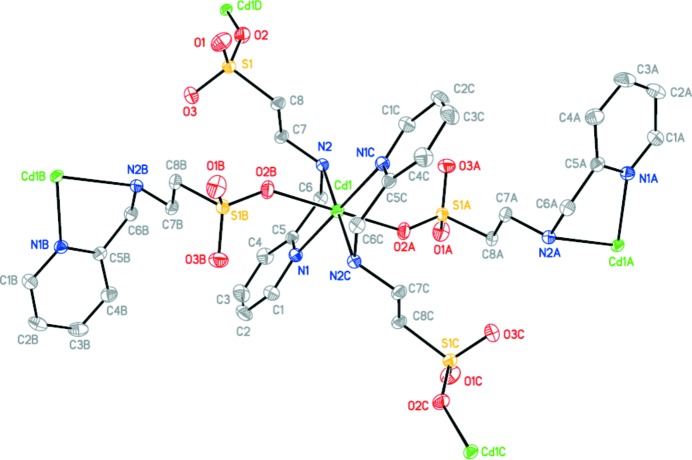
The coordination of Cd2+ ion of (I) with the atom-numbering scheme. Displacement ellipsoids drawn at the 30% probability level. H atoms have been omitted. [Symmetry codes: (A)1 - x, -1/2 + y, 0.5 - z; (B)x, 0.5 - y, -1/2 + z; (C)1 - x, -y, -z; (D)1 - x, 1/2 + y, 0.5 - z]

**Fig. 2.**
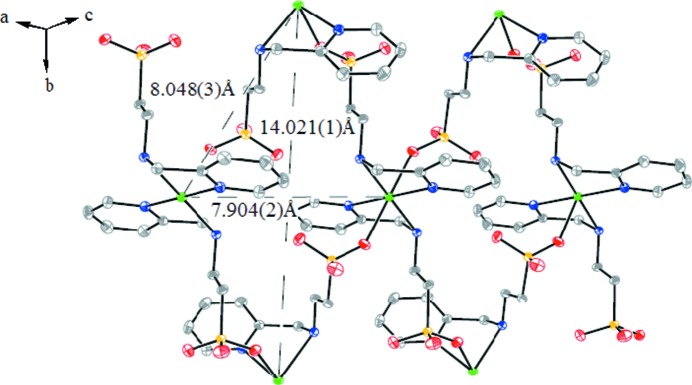
The two-dimensional layer structure of (I). H atoms have been omitted.

**Fig. 3.**
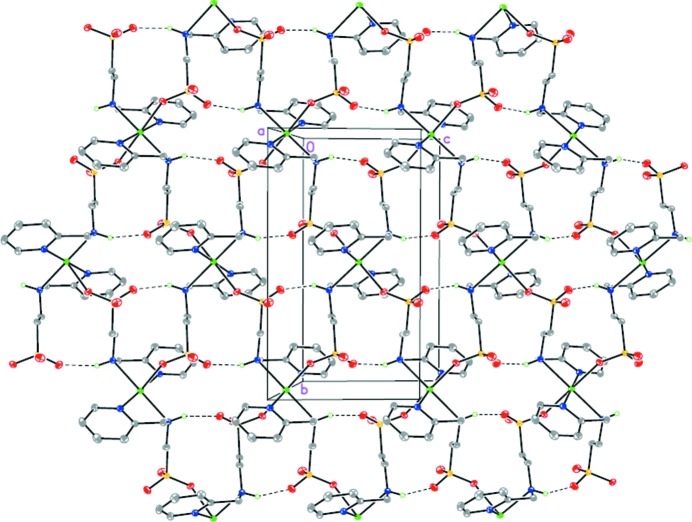
The hydrogen bonding interactions in (I)(dashed lines) projected in the plane bc. H atoms on C atoms have been omitted.

**Fig. 4.**
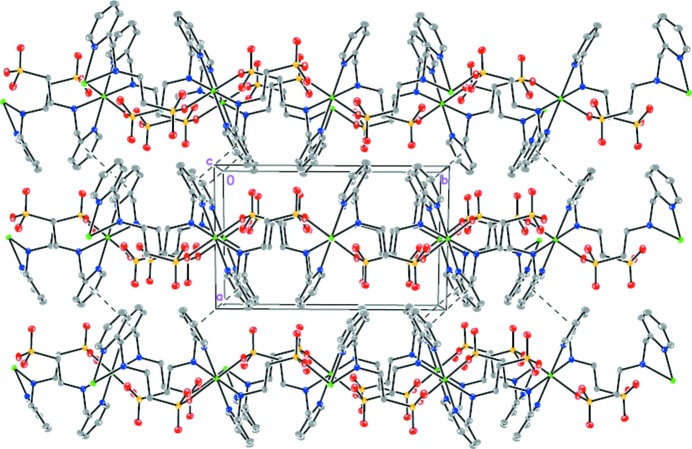
Packing diagram for (I), showing π-π stacking as dashed lines in the plane ab. H atoms on C have been deleted.

## supplementary crystallographic information

### Comment

In the previous literatures, several complexes derived from Hpmt have been reported. Among them, pmt^-^ ligand displays different coordination modes, such as tridentate chelate (Du & Zhang, 2009; Li *et al.*, 2006, 2008*a*; Liao *et al.*, 2007), µ_2_ bridge(Li *et al.*, 2007*a*, 2008*b*) and µ_3_ bridge (Li *et al.*, 2007*b*). In this paper, we describe another new complex of pmt^-^, (I), Figure 1.

Compound 1 is a two-dimensional coordination polymer and the repeating unit comprises one Cd^2+^ ion and two pmt^-^ ligands(Fig. 1, Table 1). Cd^2+^ ion situates on a centre of symmetry and is six-coordinated with four N atoms from two pmt^-^ ligands along with two sulfonate O atoms belonging to another two ligands, showing a distorted octahedral geometry (Table 1). The four N atoms from two pmt^-^ ligands define the equatorial plane with the Cd centre located in the plane, and two O atoms are at the axial positions with O2A—Cd1—O2B angle of just 180°[Symmetry codes: (A)1 - *x*, -1/2 + *y*, 0.5 - *z*; (B)*x*, 0.5 - *y*, -1/2 + *z*]. Each pmt^-^ plays as a µ_2_ bridge to connect two Cd^2+^ ions and each Cd^2+^ ion links four pmt^-^ ligands, forming an infinite two-dimensional layer structure with (4, 4) topology (Figure 2). The network is based on (Cd(pmt))_4_ rhombus, a 24-membered metal-ligand ring (–Cd—N—C—C—S—O–)_4_ formed by four pmt^-^ and four quadruply connected Cd^2+^ ions. The edge Cd···Cd distance of the rhombus is 8.048 (3)Å and the Cd···Cd separations through the diagonal of the rhombus are 7.904 (2)Å and 14.021 (1) Å, respectively (Figure 2).

The two-dimensional layer is stabilized by intermolecular N—H···O hydrogen bonds (Figure 3 & Table 2). The interlayers are further connected by π-π stacking interactions of parallel pyridine rings of adjacent layers. The interplanar average distance and ringcentroid separation distance are 3.582 (1)Å and 3.634 (1) Å, respectively. Thus, the three-dimensional supramolecular architecture of (I) is formed (Figure 4).

### Experimental

The ligand Hpmt was prepared according to the method of Li *et al.*, 2006. A water solution (5 ml) of ligand Hpmt (2 mmol, 0.432 g) was added dropwise to a solution of CdCl_2_. 2.5H_2_O (0.228 g, 1.0 mmol) in methanol (8 ml), then the obtained mixture was stirred at 333 K for 3 h. After that, the mixture was basified with KOH (1 mol/l) to a pH of 7.5–8.0 and continued stirring for another 4 h, filtrated. Two weeks later, colourless claviform crystals were grown from the filtrate by slow evaporation. Analysis, found: C 35.40, H 4.02, N 10.36, S 11.36%; C_16_H_22_CdN_4_O_6_S_2_ requires: C 35.36, H 4.05, N 10.31, S 11.42%. IR (KBr, ν, cm^-1^): 771.2[γ(CC—H)], 745.7(γCH_2_); 1188.3, 1157.4, 1040.6(ν SO_3_^-^); 1607.7, 1571.7(ν CC + ν CN); 3266.2(ν N—H). CCDC 614219.

### Refinement

H atoms bonded to C were positioned geometrically with C—H distance of 0.93–0.97 Å, and treated as riding atoms, with *U*_iso_(H)=1.2*U*_eq_(C). The N—H hydrogen atom was located in a difference Fourier map and refined isotropically.

### Crystal data

**Table:**

[Cd(C_8_H_11_N_2_O_3_S)_2_]	*F*(000) = 548
*M**_r_* = 542.90	*D*_x_ = 1.881 Mg m^−^^3^
Monoclinic, *P*2_1_/*c*	Mo *K*α radiation, λ = 0.71073 Å
Hall symbol: -P 2ybc	Cell parameters from 3852 reflections
*a* = 8.8982 (8) Å	θ = 2.4–28.2°
*b* = 14.0206 (12) Å	µ = 1.40 mm^−^^1^
*c* = 7.9040 (7) Å	*T* = 291 K
β = 103.579 (1)°	Claviform, colourless
*V* = 958.52 (15) Å^3^	0.22 × 0.16 × 0.12 mm
*Z* = 2	

### Data collection

**Table:**

Bruker APEXII CCD area-detector diffractometer	2178 independent reflections
Radiation source: fine-focus sealed tube	2008 reflections with *I* > 2σ(*I*)
graphite	*R*_int_ = 0.011
φ and ω scans	θ_max_ = 27.5°, θ_min_ = 2.4°
Absorption correction: multi-scan (*SADABS*; Sheldrick, 1996)	*h* = −7→11
*T*_min_ = 0.749, *T*_max_ = 0.849	*k* = −18→15
5691 measured reflections	*l* = −10→10

### Refinement

**Table:**

Refinement on *F*^2^	Primary atom site location: structure-invariant direct methods
Least-squares matrix: full	Secondary atom site location: difference Fourier map
*R*[*F*^2^ > 2σ(*F*^2^)] = 0.019	Hydrogen site location: inferred from neighbouring sites
*wR*(*F*^2^) = 0.049	H-atom parameters constrained
*S* = 1.03	*w* = 1/[σ^2^(*F*_o_^2^) + (0.0237*P*)^2^ + 0.5977*P*] where *P* = (*F*_o_^2^ + 2*F*_c_^2^)/3
2178 reflections	(Δ/σ)_max_ < 0.001
133 parameters	Δρ_max_ = 0.48 e Å^−^^3^
0 restraints	Δρ_min_ = −0.38 e Å^−^^3^

### Special details

**Table:**

Geometry. All e.s.d.'s (except the e.s.d. in the dihedral angle between two l.s. planes)are estimated using the full covariance matrix. The cell e.s.d.'s are takeninto account individually in the estimation of e.s.d.'s in distances, anglesand torsion angles; correlations between e.s.d.'s in cell parameters are onlyused when they are defined by crystal symmetry. An approximate (isotropic)treatment of cell e.s.d.'s is used for estimating e.s.d.'s involving l.s. planes.
Refinement. Refinement of *F*^2^ against ALL reflections. The weighted *R*-factor *wR* and goodness of fit *S* are based on *F*^2^, conventional *R*-factors *R* are based on *F*, with *F* set to zero for negative *F*^2^. The threshold expression of *F*^2^ > σ(*F*^2^) is used only for calculating *R*-factors(gt) *etc*. and is not relevant to the choice of reflections for refinement. *R*-factors based on *F*^2^ are statistically about twice as large as those based on *F*, and *R*- factors based on ALL data will be even larger.

### Fractional atomic coordinates and isotropic or equivalent isotropic displacement parameters (Å^2^)

**Table:**

	*x*	*y*	*z*	*U*_iso_*/*U*_eq_	
Cd1	0.5000	0.0000	0.0000	0.02456 (7)	
S1	0.33469 (5)	0.35013 (3)	0.12343 (6)	0.02701 (10)	
O1	0.17221 (17)	0.35246 (12)	0.0384 (2)	0.0474 (4)	
O2	0.36412 (18)	0.38778 (10)	0.30088 (18)	0.0382 (3)	
O3	0.43417 (19)	0.39430 (11)	0.0247 (2)	0.0446 (4)	
N1	0.72897 (17)	0.05064 (11)	−0.05239 (19)	0.0265 (3)	
N2	0.60386 (17)	0.11240 (10)	0.22587 (19)	0.0260 (3)	
H2	0.5777	0.0929	0.3250	0.031*	
C1	0.7692 (2)	0.04398 (15)	−0.2058 (2)	0.0342 (4)	
H1	0.7061	0.0096	−0.2957	0.041*	
C2	0.9010 (2)	0.08650 (17)	−0.2341 (3)	0.0409 (5)	
H2A	0.9275	0.0800	−0.3406	0.049*	
C3	0.9923 (2)	0.13852 (18)	−0.1025 (3)	0.0456 (5)	
H3	1.0805	0.1688	−0.1196	0.055*	
C4	0.9522 (2)	0.14571 (16)	0.0561 (3)	0.0393 (5)	
H4	1.0134	0.1805	0.1468	0.047*	
C5	0.8193 (2)	0.10009 (13)	0.0778 (2)	0.0270 (4)	
C6	0.7732 (2)	0.10068 (13)	0.2504 (2)	0.0289 (4)	
H6A	0.8052	0.0413	0.3111	0.035*	
H6B	0.8260	0.1525	0.3217	0.035*	
C7	0.5616 (2)	0.21429 (13)	0.1977 (2)	0.0301 (4)	
H7A	0.6054	0.2395	0.1055	0.036*	
H7B	0.6048	0.2499	0.3031	0.036*	
C8	0.3876 (2)	0.22747 (12)	0.1489 (2)	0.0289 (4)	
H8A	0.3435	0.1996	0.2386	0.035*	
H8B	0.3451	0.1940	0.0409	0.035*	

### Atomic displacement parameters (Å^2^)

**Table:**

	*U*^11^	*U*^22^	*U*^33^	*U*^12^	*U*^13^	*U*^23^
Cd1	0.02285 (10)	0.02064 (10)	0.03077 (10)	−0.00396 (6)	0.00747 (7)	−0.00131 (7)
S1	0.0320 (2)	0.0237 (2)	0.0260 (2)	0.00119 (16)	0.00802 (17)	−0.00138 (16)
O1	0.0355 (8)	0.0447 (9)	0.0561 (10)	0.0066 (6)	−0.0011 (7)	−0.0040 (7)
O2	0.0547 (9)	0.0275 (7)	0.0328 (7)	−0.0018 (6)	0.0109 (6)	−0.0074 (6)
O3	0.0567 (9)	0.0389 (8)	0.0447 (8)	0.0005 (7)	0.0249 (7)	0.0101 (7)
N1	0.0246 (7)	0.0267 (8)	0.0287 (7)	−0.0016 (6)	0.0071 (6)	0.0000 (6)
N2	0.0295 (7)	0.0237 (7)	0.0263 (7)	−0.0002 (6)	0.0098 (6)	0.0002 (6)
C1	0.0332 (10)	0.0393 (11)	0.0309 (9)	0.0007 (8)	0.0092 (8)	−0.0007 (8)
C2	0.0353 (10)	0.0545 (13)	0.0375 (10)	0.0039 (9)	0.0178 (9)	0.0075 (10)
C3	0.0289 (10)	0.0554 (14)	0.0563 (14)	−0.0057 (9)	0.0176 (9)	0.0085 (11)
C4	0.0274 (9)	0.0428 (11)	0.0468 (12)	−0.0094 (8)	0.0071 (8)	−0.0037 (9)
C5	0.0234 (8)	0.0245 (8)	0.0324 (9)	0.0015 (6)	0.0052 (7)	0.0018 (7)
C6	0.0271 (9)	0.0294 (9)	0.0277 (8)	−0.0013 (7)	0.0018 (7)	−0.0015 (7)
C7	0.0336 (9)	0.0217 (8)	0.0361 (9)	−0.0005 (7)	0.0103 (8)	−0.0035 (7)
C8	0.0332 (9)	0.0226 (8)	0.0316 (9)	−0.0006 (7)	0.0089 (7)	−0.0056 (7)

### Geometric parameters (Å, °)

**Table:**

Cd1—N1	2.2853 (14)	C1—C2	1.380 (3)
Cd1—N1^i^	2.2853 (14)	C1—H1	0.9300
Cd1—O2^ii^	2.3496 (14)	C2—C3	1.370 (3)
Cd1—O2^iii^	2.3497 (14)	C2—H2A	0.9300
Cd1—N2	2.3979 (15)	C3—C4	1.385 (3)
Cd1—N2^i^	2.3979 (15)	C3—H3	0.9300
S1—O1	1.4447 (15)	C4—C5	1.390 (3)
S1—O3	1.4496 (15)	C4—H4	0.9300
S1—O2	1.4635 (14)	C5—C6	1.514 (2)
S1—C8	1.7821 (18)	C6—H6A	0.9700
O2—Cd1^iv^	2.3497 (14)	C6—H6B	0.9700
N1—C5	1.341 (2)	C7—C8	1.516 (3)
N1—C1	1.346 (2)	C7—H7A	0.9700
N2—C7	1.481 (2)	C7—H7B	0.9700
N2—C6	1.483 (2)	C8—H8A	0.9700
N2—H2	0.9100	C8—H8B	0.9700
			
N1—Cd1—N1^i^	180	N1—C1—H1	118.9
N1—Cd1—O2^ii^	89.37 (5)	C2—C1—H1	118.9
N1^i^—Cd1—O2^ii^	90.63 (5)	C3—C2—C1	118.77 (19)
N1—Cd1—O2^iii^	90.63 (5)	C3—C2—H2A	120.6
N1^i^—Cd1—O2^iii^	89.37 (5)	C1—C2—H2A	120.6
O2^ii^—Cd1—O2^iii^	179.999 (1)	C2—C3—C4	119.59 (19)
N1—Cd1—N2	74.13 (5)	C2—C3—H3	120.2
N1^i^—Cd1—N2	105.87 (5)	C4—C3—H3	120.2
O2^ii^—Cd1—N2	83.89 (5)	C3—C4—C5	119.02 (19)
O2^iii^—Cd1—N2	96.11 (5)	C3—C4—H4	120.5
N1—Cd1—N2^i^	105.87 (5)	C5—C4—H4	120.5
N1^i^—Cd1—N2^i^	74.13 (5)	N1—C5—C4	121.17 (17)
O2^ii^—Cd1—N2^i^	96.11 (5)	N1—C5—C6	116.99 (15)
O2^iii^—Cd1—N2^i^	83.89 (5)	C4—C5—C6	121.80 (17)
N2—Cd1—N2^i^	180.0	N2—C6—C5	111.41 (14)
O1—S1—O3	114.25 (10)	N2—C6—H6A	109.3
O1—S1—O2	111.86 (9)	C5—C6—H6A	109.3
O3—S1—O2	111.57 (9)	N2—C6—H6B	109.3
O1—S1—C8	106.47 (9)	C5—C6—H6B	109.3
O3—S1—C8	107.12 (9)	H6A—C6—H6B	108.0
O2—S1—C8	104.88 (9)	N2—C7—C8	111.36 (15)
S1—O2—Cd1^iv^	146.26 (9)	N2—C7—H7A	109.4
C5—N1—C1	119.27 (16)	C8—C7—H7A	109.4
C5—N1—Cd1	114.90 (11)	N2—C7—H7B	109.4
C1—N1—Cd1	125.37 (13)	C8—C7—H7B	109.4
C7—N2—C6	109.95 (14)	H7A—C7—H7B	108.0
C7—N2—Cd1	118.81 (11)	C7—C8—S1	111.93 (12)
C6—N2—Cd1	103.10 (10)	C7—C8—H8A	109.2
C7—N2—H2	108.2	S1—C8—H8A	109.2
C6—N2—H2	108.2	C7—C8—H8B	109.2
Cd1—N2—H2	108.2	S1—C8—H8B	109.2
N1—C1—C2	122.17 (19)	H8A—C8—H8B	107.9
			
O1—S1—O2—Cd1^iv^	−124.02 (16)	Cd1—N1—C1—C2	171.82 (15)
O3—S1—O2—Cd1^iv^	5.3 (2)	N1—C1—C2—C3	−1.1 (3)
C8—S1—O2—Cd1^iv^	120.97 (16)	C1—C2—C3—C4	1.3 (3)
O2^ii^—Cd1—N1—C5	−71.71 (12)	C2—C3—C4—C5	−0.3 (3)
O2^iii^—Cd1—N1—C5	108.29 (12)	C1—N1—C5—C4	1.0 (3)
N2—Cd1—N1—C5	12.12 (12)	Cd1—N1—C5—C4	−171.65 (15)
N2^i^—Cd1—N1—C5	−167.88 (12)	C1—N1—C5—C6	−176.72 (16)
O2^ii^—Cd1—N1—C1	116.16 (16)	Cd1—N1—C5—C6	10.6 (2)
O2^iii^—Cd1—N1—C1	−63.84 (16)	C3—C4—C5—N1	−0.9 (3)
N2—Cd1—N1—C1	−160.01 (16)	C3—C4—C5—C6	176.77 (19)
N2^i^—Cd1—N1—C1	19.99 (16)	C7—N2—C6—C5	−80.67 (18)
N1—Cd1—N2—C7	90.73 (12)	Cd1—N2—C6—C5	46.99 (15)
N1^i^—Cd1—N2—C7	−89.27 (12)	N1—C5—C6—N2	−42.0 (2)
O2^ii^—Cd1—N2—C7	−178.19 (12)	C4—C5—C6—N2	140.29 (18)
O2^iii^—Cd1—N2—C7	1.81 (12)	C6—N2—C7—C8	172.59 (14)
N1—Cd1—N2—C6	−31.13 (10)	Cd1—N2—C7—C8	54.25 (18)
N1^i^—Cd1—N2—C6	148.87 (10)	N2—C7—C8—S1	177.53 (12)
O2^ii^—Cd1—N2—C6	59.95 (10)	O1—S1—C8—C7	166.69 (13)
O2^iii^—Cd1—N2—C6	−120.05 (10)	O3—S1—C8—C7	44.07 (16)
C5—N1—C1—C2	0.0 (3)	O2—S1—C8—C7	−74.60 (15)

Symmetry codes: (i) −*x*+1, −*y*, −*z*; (ii) −*x*+1, *y*−1/2, −*z*+1/2; (iii) *x*, −*y*+1/2, *z*−1/2; (iv) −*x*+1, *y*+1/2, −*z*+1/2.

### Hydrogen-bond geometry (Å, °)

**Table:**

*D*—H···*A*	*D*—H	H···*A*	*D*···*A*	*D*—H···*A*
N2—H2···O3^v^	0.91	2.26	3.089 (2)	152

Symmetry codes: (v) *x*, −*y*+1/2, *z*+1/2.
